# P-1812. Artificial Intelligence Modeling and Implementation in Antimicrobial Stewardship Program at a Tertiary Medical Center in Taiwan

**DOI:** 10.1093/ofid/ofae631.1975

**Published:** 2025-01-29

**Authors:** Chun-Wen Cheng, Po-Yen Huang, Hsuan-Ling Hsiao, Jung-Sheng Chen, Cheng-Hsun Chiu, Chang-Fu Kou

**Affiliations:** Chang Gung Memorial Hospital at Lin-kou, Taoyuan, Taiwan., Gueishan, Taoyuan, Taiwan (Republic of China); Chang Gung Memorial Hospital, Taoyuan, Taiwan., Gueishan, Taoyuan, Taiwan; Chang Gung Memorial Hospital, Taoyuan, Taiwan, Gueishan, Taoyuan, Taiwan; Chang Gung Memorial Hospital, Taoyuan, Taiwan, Gueishan, Taoyuan, Taiwan; Chang Gung Memorial Hospital, Kweishan, Taoyuan, Taiwan; Chang Gung Memorial Hospital, Taoyuan, Taiwan, Gueishan, Taoyuan, Taiwan

## Abstract

**Background:**

Antimicrobial stewardship program (ASP) aim to promote appropriate antibiotic use and preventing antimicrobial resistance. Personalized tools using artificial intelligence (AI) to provide tailored antibiotic recommendations to healthcare providers may transform the way we approach antibiotic stewardship.

**Figure 1:**
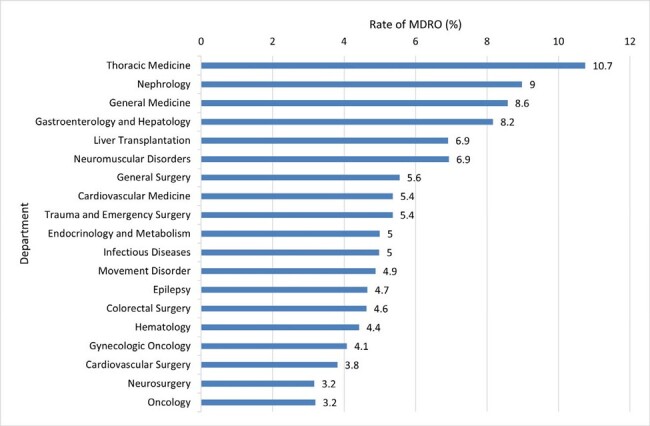
Rate of Multidrug-Resistant Organisms by Department

**Methods:**

The study included all patients (age > 12) admitted to Chang Gung Memorial Hospital from January 1, 2005, through January 31, 2022, who had been prescribed antibiotics after admission and had microbiological evidence within the hospitalization. We conducted a linear regression equation to explore the relationship between antibiotic heterogeneity index (AHI) and multidrug-resistant organisms (MDROs) ratio in different units of hospital. We used a variety of machine learning algorithms to evaluate the incremental value of various variable groups of patients, and developed an AI model for predicting MDRO infections and antimicrobial de-escalation during hospitalization.

**Figure 2:**
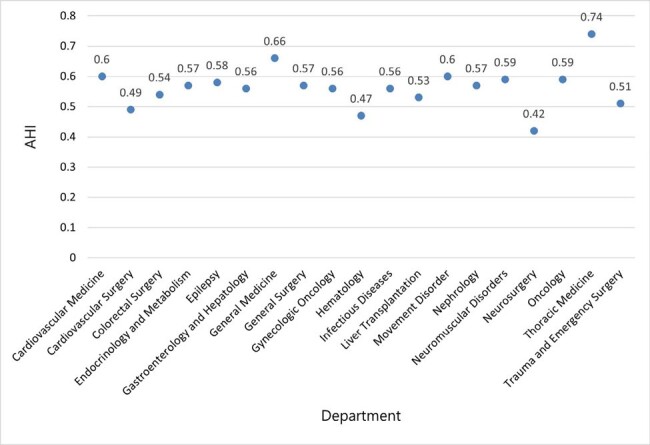
Value of Antibiotic Heterogeneity Index by Department AHI: antimicrobial heterogenecity index

**Results:**

There were 789,259 individuals fulfilled the including criteria. Figure 1 shows the incidence rates of MDROs across 19 departments, ranging from 3.2% in oncology to 10.7% in thoracic medicine. AHI for each department was ranging from 0.42 in neurosurgery to 0.74 in thoracic medicine (figure 2). Linear regression for the relationship between AHIs and MDRO incidence rates, represented a regression coefficient β of 0.179 (p-value: 0.02), indicating a statistically significant difference, with an R-squared of 0.47. Variables considered in modeling analysis of MDRO infection and antimicrobial de-escalation were shown in table 3. Random Forests with model 3 variables had the best (97%) positive predictive value (PPV) among five machine learning model in predicting MDRO infection, and the performance is much better than traditional linear regression (Figure 4). However, the best predicting model for antimicrobial de-escalation had only 43% PPV in Random Forests with model 5 variables.

**Table 3:**
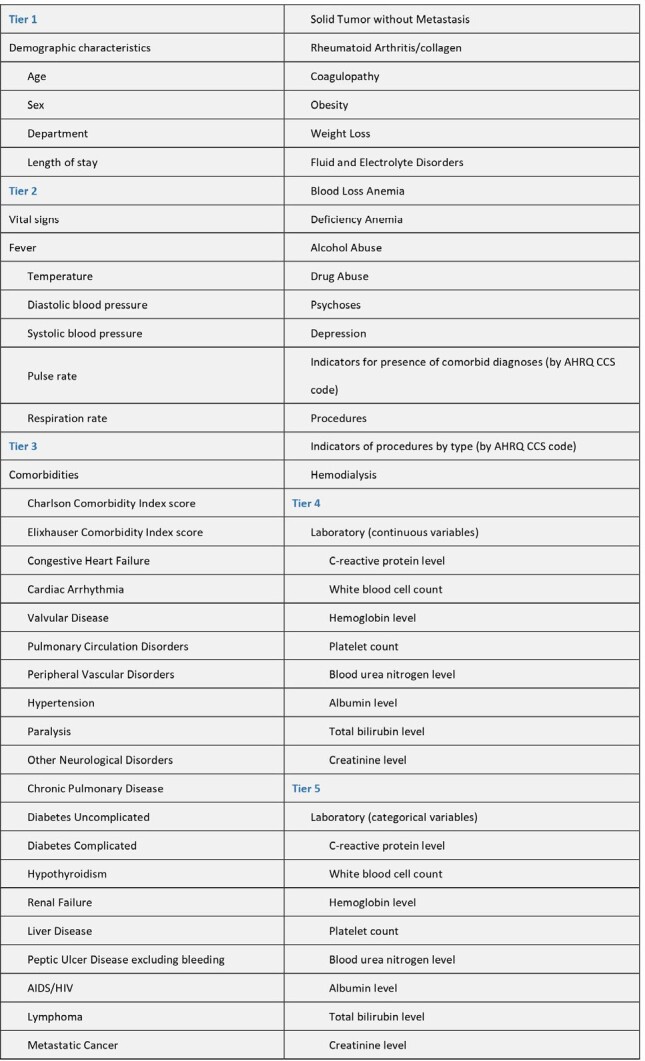
Variables Considered in Modeling Analyses of Multidrug-Resistant Organisms and Antibiotic De-escalation Tiered on Feasibility of Measurement

**Conclusion:**

High AHI had positive correlation with high MDRO incidence. AI modeling had a great performance in predicting MDRO infection. Early predicted high risk group can be an important special population for ASP implementation.

**Figure 4:**
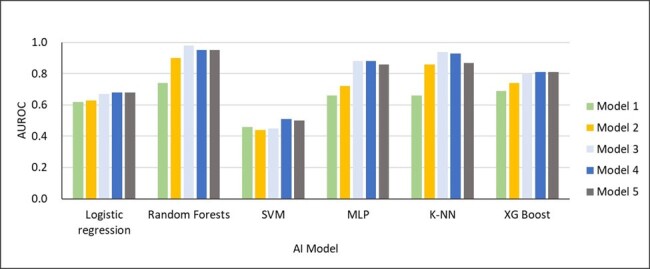
Model Performance of Classification for Multidrug-Resistant Organisms by Input Variable Feasibility Tier

Model 1 included tier 1 factors as predictor variables to predict interested outcome; model 2 included both tier1 and tier2 as predictor variables; model 3 use tier1 to tier3 as predictor variables; model 4 included tier1 to tier3 variables and plus tier 4 factors which contains continuous laboratory variables as predictor variables and model 5 included tier1 to tier3 variables and plus tier 5 factors which contains categorical laboratory variables as predictor variables.

**Disclosures:**

**All Authors**: No reported disclosures

